# Spectrophotometric determination of creatinine by monosegmented continuous-flow analysis

**DOI:** 10.1155/S146392469200021X

**Published:** 1992

**Authors:** Lourival C. de Faria, Celio Pasquini

**Affiliations:** Instituto de Química Universidade Estadual de Campinas Caixa Postal 6154 Campinas, SP CEP 13081 Brazil

## Abstract

A monosegmented continuous-flow system (MCFS) has been evaluated for determination of creatinine in urine using the Jaffé reaction. The analyser is compact and allows 130 determinations to be performed per hour, with a relative standard deviation of the peak height better than 1.5% (N =10). The results for real samples agree with those obtained by. the standard manual Jaffé procedure and with the kinetic automatic method.


Journal of Automatic Chemistry, Vol. 14, No. 3 (May-June 1992), pp. 97-100

### Spectrophotometric determination of creatinine by monosegmented continuous-flow analysis

**Lourival C. de Faria and Celio Pasquini**

*Instituto de Química, Universidade Estadual de Campinas, Caixa Postal 6154, CEP 13081, Campinas, SP, Brazil*

*A monosegmented continuous-flow system (MCFS) has been evaluated for determination of creatinine in urine using the Jaffé reaction. The analyser is compact and allows 130 determinations to be performed per hour, with a relative standard deviation of the peak height better than 1·5% (N = 10). The results for real samples agree with those obtained by the standard manual Jaffé procedure and with the kinetic automatic method.*

### Introduction

Creatinine in urine is often tested in clinical laboratories to detect kidney disease. Although a number of methodologies have been proposed for creatinine determination [1-4], the Jaffé reaction [5 and 6] is the one most often used in clinical laboratories. This methodology is based on the formation of a red-to-orange compound, which is generated by the reaction of the creatinine with picric acid in an alkaline medium. The method has been automated for the Technicon multisegmented continuous flow analyser [7,8] and for such discrete analysers as the Cobas-Mira. Flow Injection Analysis (FIA) systems have been also described [9-11], but they are based on more complex detection techniques [10-11]. The Jaffé reaction requires the use of devices like heat baths or long reaction coils, to overcome the lack of sensitivity due to the low residence time allowed by the FIA system [9].

Monosegmented Continuous Flow Analysis (MCFA) has been recently introduced [12 and 13] as an alternative to FIA when long sample residence times are required in methodologies involving slow chemical reactions. In MCFA the sample is introduced into the incubation coil between two air bubbles. Sample dispersion is, therefore, restricted and the residence time can be as large as 5 min (keeping the sampling rate as high as 120 samples per hour) [12]. MCFA uses the same equipment as FIA and results in very simple and versatile manifolds when compared with Technicon AutoAnalyzer. Due the long residence times that can be achieved by the MCFS, the system appears to be suitable for application in clinical chemistry. This work describes the use of an MCFS for determining creatinine in urine.

### Experimental

### *Reagents*

Stock creatinine solution was prepared by dissolving 1·0000 g of creatinine PA in 1 l of 0·1 M HCl solution. Standard solutions, from 3 to 15 mg/l, were obtained by suitable dilution of the stock solution. Picric acid solution (0·04 M) was prepared by dissolving 9·2 g of picric acid in 500 ml of water at 80 °C. The solution was cooled to ambient temperature and the volume was taken to 1 l. The concentration was checked by titration with 0·1 M NaOH using phenolphthalein as indicator. All other reagents were of PA analytical grade.

### *Samples*

Urine samples were supplied by the State University of Campinas's hospital, 1 ml of sample was diluted with deionized water to 250 ml and 300 μl were introduced in the monosegmented manifold.

### *Monosegmented manifold*

The monosegmented manifold shown in figure 1 was used for the creatinine determination. The manifold removes air bubbles mechanically, as previously described [13], and employs two sampling valves.

The sample and reagents are mixed continuously before valve 1 and then fed to its sampling loop, L1 (300 μl). Simultaneously, the two small loops (30 μl) are filled with air by suction through point E, using a water-aspirator. When the central part of valve 1 is moved, the two air loops and the sampling loop (which contains the sample mixed with reagents) are placed in the carrier line. The carrier, C1, usually an inert fluid, impels the air-sample-air sequence to valve 2 passing through the incubation coil I. The carrier flow rate and the length of the coil I determine the residence time (and thus the time available for the reaction to process) in the system. To reduce the size of the coil I, the carrier flow rate is kept at the minimum necessary to introduce the monosegment in the coil and to provide enough carrier fluid to avoid any sample inter-contamination (carry-over). Various monosegments are present at the same time in the incubation coil. The air bubbles prevent axial dispersion and the carrier fluid in between segments washes the thin fluid film left behind by the samples.

At the end of the incubation coil, the monosegment reaches valve 2. When the resampling loop, L2 (150 μl), is filled with the sample and the air bubbles are out (one has passed through valve 2 and the other is still in the incubation coil), the central portion is moved and the reacted sample is introduced in a parallel carrier line which transports the sample, free of air bubbles, to the flow cell. The path from valve 2 to the flow cell is made to be as small as possible in order to avoid sample dispersion and loss in sensitivity because the sample is at this stage

0142-0453/92 $3.00 © 1992 Taylor & Francis Ltd.

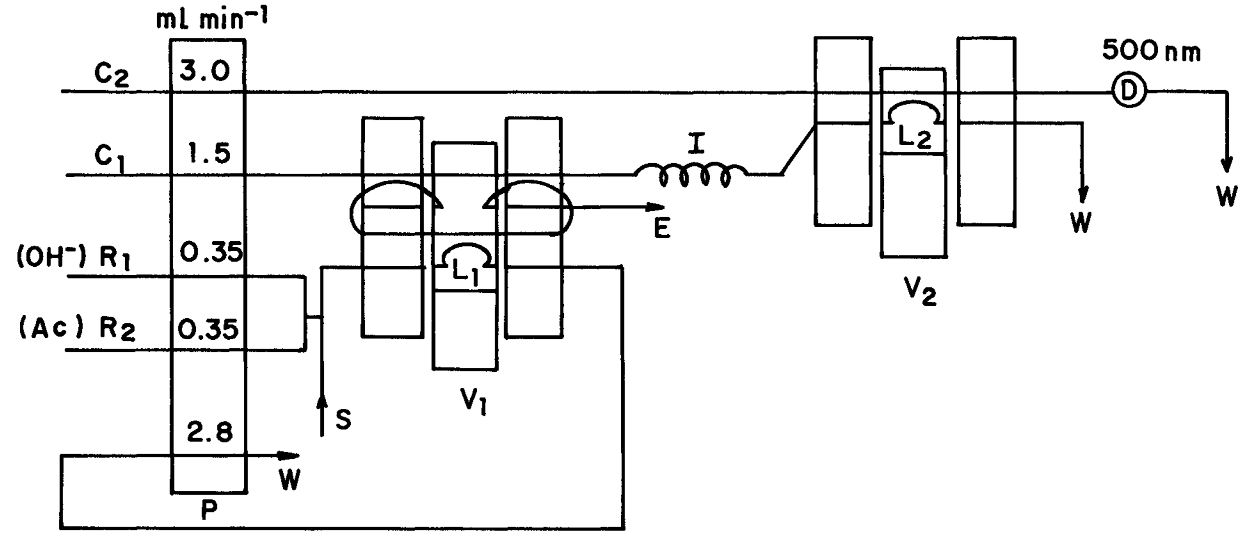

*Figure 1. Monosegmented Continuous Flow Analysis manifold for the determination of creatinine. P, peristaltic pump; V1, sample introduction valve; S, sample inlet; W, waste line; E, water aspirator; I, incubation coil; V2, resample, air bubble removing valve; D, Detector; L1, sample monosegment loop (300 μl); L2, resample loop (150 μl); C1 and C2, 1% (m/v) NaCl carrier streams.*

being carried in an unsegmented mode like an FIA system. The role of the carrier C2 is simply to transport the sample to the detector. A high flow rate is employed to rapidly wash the flow cell. Therefore, the manifold described in figure 1 is capable of optimizing the analyser design by allowing the incubation coil to be sized independently of the sample washing time, as this is defined in the parallel line C2.

The operation of the valves must be synchronized. When the manifold is first employed, the user should, with the aid of a stopwatch, find the time intervals at which the central part of the valves must be switched. The movement of each valve is accomplished by the use of two solenoids operating from the mains supply. The interface between the single board microcomputer and the solenoids is made, as previously described [14], employing a CI 8155 I/O peripheral device and a BC428 transistor that switches an electromagnetic relay which actuates the mains operated solenoids. The software, written in machine code for the 8085 CPU, controls the time intervals at which the valves remain in one or other position.

After the synchronous condition is achieved, which is important mainly for the air removal, the system will operate accurately for a long period of time. The critical parameter to be kept constant during operation is the flow rate of the carrier C1. However, as only 150 μl of the 300 μl monosegment is resampled in valve 2, some change in the C1 flow rate can be tolerated without a risk of introducing an air bubble in the detector line.

Concerning the creatinine determination, a residence time of 240 s was found to be enough to reach a suitable sensitivity for the determination in urine. Therefore, a 50 cm long, 4 mm inner diameter glass tube coiled with 5 cm diameter was used for the incubation path.

A peristaltic pump, Ismatec MP13 GJ4, provided with Tygon pumping tubes was employed to impel the fluids.

A Zeiss PM2D spectrophotometer, furnished with a 1 cm optical path, 80 μl flow cell, was used for the detection of the product colour absorbance at 500 nm. The analytical signals were recorded by a ECB RE101 potentiometric recorder.

The effect of the concentration of the sodium hydroxide reagent solution was determined in the range from 0·5 to 2·0 M. In this range, an increase of only 15% in the peak height was observed for creatinine standard solutions of 6 and 12 mg/l when the most concentrated reagent solution was used. However, at this concentration the sodium picrate tends to precipitate in the manifold and the reproducibility of the signals is poor. Best results, in terms of reproducibility and sensitivity, were obtained at an NaOH concentration equal to 0·76 M. The best concentration of picric acid was found to be 0·040 M, which is enough to reach the maximum peak height in the manifold. To avoid refractive index effects in the detection process a 1% (m/v) NaCl solution was employed as a carrier stream in both the incubation and detector lines.

### Results and discussion

Figure 2 shows the calibration signals and sample signals obtained for creatinine determination employing the monosegmented manifold described in figure 1. The sampling frequency was 130/h and the signals presented a relative standard deviation lower than 1·5% for 10 replicates of 6 and 12 mg/l creatinine standard solutions.

Ten urine samples were determined for their creatinine content using the manual Jaffé procedure, the discrete automated Cobas-Mira kinetic Jaffé procedure [15], and the MCFS. The results are shown in table 1. Statistical

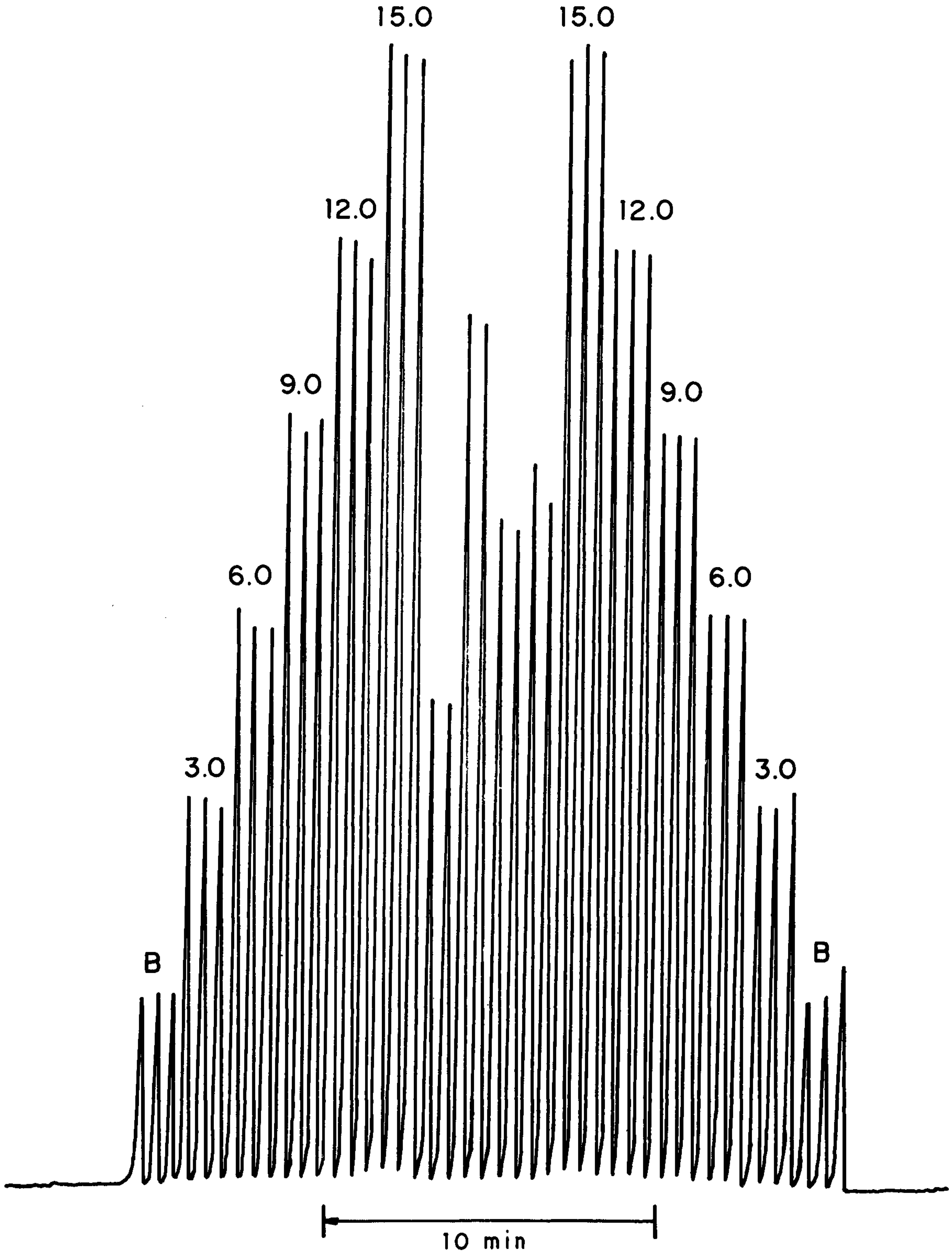

*Figure 2. Analytical signals for creatinine determination. B indicates a blank proof and the numbers over the signals indicate the creatinine concentration in mg/l. From the right, the blank and five standard solutions were introduced in triplicate, four urine samples were introduced in duplicate, and then the standard and blank were introduced again in triplicate.*

comparison of the 10 results shows that the creatinine concentration found by the present method (Cp in mg/dl) is related to the Cobas-Mira kinetic results (Cm) by:

$$Cp = (0{\cdot}388 \pm 0{\cdot}743) + (0{\cdot}9415 \pm 0.0208)Cm$$

with a correlation coefficient of 0·9980; the standard error of the estimate is ± 0·256 mg/dl. No significant difference was observed among the three methods at the 95% confidence level.

The MCFA method for creatinine determination has several advantages over the FIA method previously described [9]. A long incubation time (240 s) can be achieved with only a small decrease in sensitivity and at

*Table 1. Comparative results obtained for determination of creatinine in urine samples by MCFS and by two different standard methods.*

| Sample | Creatinine (mg/dl) Manual method | Automatic kinetic | MCFS |
|---|---|---|---|
| 1 | 3·40 | 3·49 | 3·44 |
| 2 | 14·71 | 14·98 | 16·00 |
| 3 | 8·04 | 8·32 | 8·56 |
| 4 | 8·29 | 8·25 | 8·15 |
| 5 | 7·53 | 7·62 | 7·51 |
| 6 | 10·88 | 10·79 | 10·76 |
| 7 | 4·59 | 4·61 | 4·62 |
| 8 | 7·21 | 7·32 | 7·25 |
| 9 | 13·20 | 13·04 | 13·13 |
| 10 | 3·33 | 3·44 | 3·40 |

good sample throughput (130 samples/h). No heating bath is required and the sample dilution overcomes any colour matrix effect. The size of the reaction coil can be reduced from 1740 cm to 30 cm, resulting in a more compact analyser. The concentration of NaOH is lower than that required for the FIA system [9]. Keeping the NaOH concentration low helps to improve the base-line stability because it does not promote the formation of picrate precipitate. Also, sample and reagent consumption is low and an overall evaluation shows that the MCFS can be used in creatinine determination with the same performance as other automation techniques.

### Acknowledgements

The authors are grateful to the Clinical Analysis Laboratory of the State University of Campinas for supplying the urine samples.

### References

1. Benedict, S. R. and Behre, J. A., *Journal of Biological Chemistry*, **114** (1936), 515.
2. Langley, W. D. and Evans, M., *Journal of Biological Chemistry*, **115** (1936), 457.
3. Miller, B. F. and Dubos, R., *Journal of Biological Chemistry*, **121** (1937), 457.
4. Sullivan, M. X. and Irreverre F., *Journal of Biological Chemistry*, **233** (1958), 530.
5. Jaffé, M., *Z. Physiol. Chem.*, **10** (1886), 391.
6. Folin, O., *Z. Physiol. Chem.*, **41** (1904), 223.
7. Jansen, A. P., Peters, K. A. and Zelders, T., *Clinica Chimica Acta*, **27** (1970), 125.
8. Moll, M. and Pring, B. A., *Journal of Clinical Pathology*, **24** (1971), 88.
9. van Staden, J. F., *Fresenius Z. Anal. Chem.*, **315** (1983), 141.
10. Winquist, F., Lundstrom, I. and Danielsson, B., *Analytical Chemistry*, **58** (1986), 145.
11. Petterson, B. A., Hansen, E. H. and Ruzicka, J., *Analytical Letters*, **19** (1986), 649.
12. Pasquini, C. and de Oliveira, W. A., *Analytical Chemistry*, **57** (1985), 2575.
13. Pasquini, C., *Analytical Chemistry*, **58** (1986), 2346.
14. Pasquini, C. and de Faria, L. C., *Journal of Automatic Chemistry*, **13** (1991), 143.
15. *Cobas-Mira Operation and User Manual* (1989).

